# The Development of Sun-Tracking System Using Image Processing

**DOI:** 10.3390/s130505448

**Published:** 2013-04-24

**Authors:** Cheng-Dar Lee, Hong-Cheng Huang, Hong-Yih Yeh

**Affiliations:** High Concentration Photovoltaic R&D Project, Institute of Nuclear Energy Research, Taoyuan 32546, Taiwan; E-Mails: hwhuang@iner.gov.tw (H.-C.H.); markyeh@iner.gov.tw (H.-Y.Y.)

**Keywords:** image-based Sun position sensor, image processing, four-quadrant light sensor, bar-shadow photo sensor

## Abstract

This article presents the development of an image-based sun position sensor and the algorithm for how to aim at the Sun precisely by using image processing. Four-quadrant light sensors and bar-shadow photo sensors were used to detect the Sun's position in the past years. Nevertheless, neither of them can maintain high accuracy under low irradiation conditions. Using the image-based Sun position sensor with image processing can address this drawback. To verify the performance of the Sun-tracking system including an image-based Sun position sensor and a tracking controller with embedded image processing algorithm, we established a Sun image tracking platform and did the performance testing in the laboratory; the results show that the proposed Sun tracking system had the capability to overcome the problem of unstable tracking in cloudy weather and achieve a tracking accuracy of 0.04°.

## 1. Introduction

Solar power is in demand and its use has been growing fast in the recent decades because of climate changing issues and the depletion of fossil fuels. Though the European debt crisis has led to weaker growth of the World economy, and even economic downturns for some countries, in addition to the weakened demand for solar energy as a result of lower European country government subsidies, solar power could be promoted again once the price without subsidies is reasonable. The European Photovoltaic Industry Association (EPIA) forecast that PV systems could reach a Levelised Cost of Energy (LCOE) of 5–12 €cts/kWh in sunbelt countries by 2020 [1]. It would then likely be more competitive than traditional sources, such as gas or oil fuelled peak power plants. GTM Research has predicted that the LCOE for concentration photovoltaic (CPV) systems will be 0.06 $/kWh by 2020 [2].

High accuracy dual axis solar trackers, tracking the Sun's motion across the sky, are used in high concentration photovoltaic (HCPV) systems because HCPV systems can only accept direct solar light and the acceptance angle deviation from the Sun is very low for a HCPV module. Therefore, the use of highly accurate and stable solar trackers is important to obtain the maximum annual electric power for a HCPV system. In addition, the solar tracker can be applied to a PV system to produce more power than with a flat PV system. A dual axis solar tracker can provide up to 50% power more in theory [3], depending on weather and latitude, but a tracking error of a few degrees is just enough for a PV system.

Some reviews about sun-tracking methods for maximizing solar systems’ output have been published [4,5]. In those papers open-loop and closed-loop control for sun-tracking are described. As to closed-loop control, a Sun position sensor is used to provide feedback signals to judge where the Sun is. Many papers describe the design of Sun position sensors [6–10]. Most of these designs are bar-shadow type photosensors. One shadow bar is located at the center of the sensor mechanism and four photodiodes are separated at the four cardinal points around the shadow bar. The solar tracker continues to track the Sun until the difference between the east and west photosensor signal is less than some threshold and the difference between the north and south photosensor signal is less than a threshold, thus, the solar tracker is completely aimed at the Sun. Bar-shadow sun sensors can maintain high accuracy in sunny days, but they don’t on cloudy days because of the lower sensitivity. Furthermore, characteristic mismatch among the four photodiodes is also a problem. Consequently, the generation power of a PV system would decrease due to these factors. Afterwards four-quadrant light sensors were applied in Sun tracking [11,12]. The use of four-quadrant light sensors with a pinhole mechanism improves the characteristic mismatch of bar-shadow photosensors but it is still affected by low irradiation and consequently results in lower PV system power output.

In order to solve the problems stated above, an image-based Sun position sensor has been developed in recent years. A charge-couple device (CCD) is used to detect the Sun's image for aerospace missions [13]. An optical head with multiple apertures and a CMOS photodetector are combined to form an image-based Sun position sensor [14]. This allows simultaneous multiple acquisitions of the Sun as spots on the focal plane. The advantage if the design is the enhanced accuracy when pointing to the Sun. As described in the context, the average sensor accuracy is better than 0.01°. A sensor composed of a commercial plug-and-play webcam and a polarized filter is presented in [15]. It proved that the image-based Sun position sensor has high immunity to different weather conditions and achieved a tracking accuracy of 0.1°.

The stability of the solar tracking system is a key factor to obtain the maximum electric power from a PV system. We have developed an image-based Sun position sensor to increase the stability and accuracy of Sun-tracking. The image-based Sun position sensor consists of a self-design reflecting Cassegrain telescope and webcam. The reflecting telescope can enlarge and adjust Sun pictures to an adequate size to achieve optimum tracking accuracy and view angle. This article describes the development of an image-based Sun position sensor and the algorithm for how to point at the Sun precisely by using image processing.

The paper is organized as follows: In Section 2, the design and analysis of a reflecting Cassegrain telescope are described. In Section 3, the control strategies of Sun-tracking are examined. In Section 4, the experimental results that prove the good performance of our image-based tracking system are presented. Finally, some conclusions are offered in Section 5.

## 2. Design and Analysis of a Reflecting Cassegrain Telescope

Obtaining a clear photo with a large enough Sun image is the first and determining step for accurately estimating the solar center. If the Sun image taken in the photo is too small, due to the digitizing effects, it may introduce uncharacteristic noise and errors to the image that become difficult to filter out to estimate the solar center.

**Figure 1.**
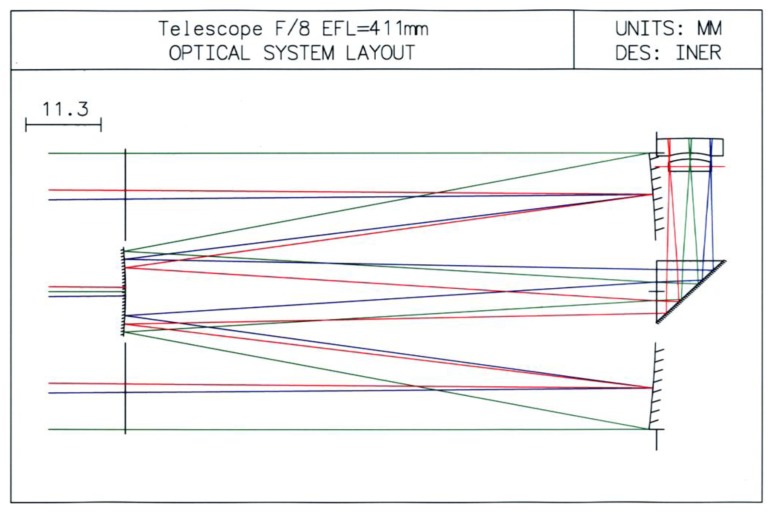
A reflecting type Cassegrain telescope–optical structure.

A reflecting type Cassegrain telescope [16,17] can reflect and enlarge Sun images. To weaken the Sun luminosity and shorten the telescope length, we put in a right angle prism to change the light direction and allocate a suitable eyepiece to get an enlarged Sun image and also a clear outline. We thus developed a Cassegrain type telescope with a right angle and eyepiece as shown in Figure 1.

In the plot, there are two concave mirrors and a right prism at the right side, and a convex mirror at the left side, as well as an eyepiece above the right prism. The two concave mirrors reflect light to convex mirror, and then the light reflected by the convex mirror goes to the right prism. Subsequently, the light is refracted to the eyepiece. Finally, the light goes through the eyepiece and focuses on somewhere above the eyepiece.

**Figure 2.**
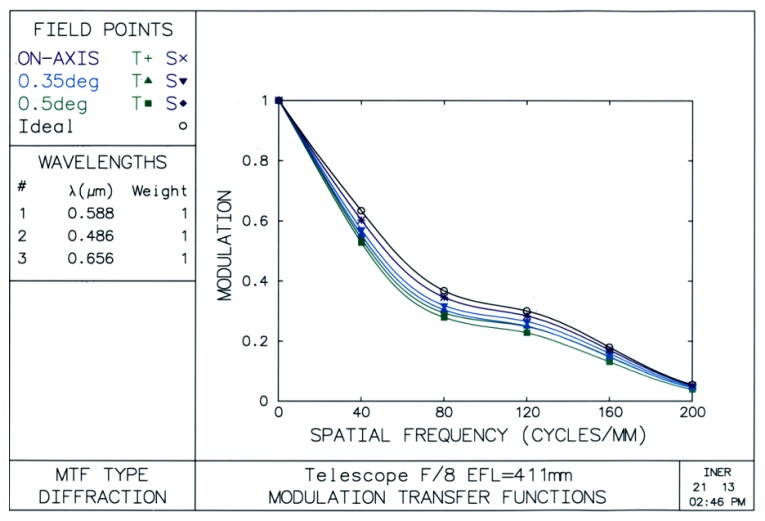
MTF analysis of a reflecting type Cassegrain telescope.

The OSLO^®^ tool [18] is used to analyze the characteristics of the self-designed telescope. In assessing performance of the telescope, as shown in Figure 2, modulation transfer functions (MTF) give an idea about how much contrast a telescope design will be able to show [19].

The MTF (modulation value) *γ*, the axis of ordinates, can be expressed as in Equation (1):

(1) $$\gamma =\frac{I_{\text{max}}-I_{\text{min}}}{I_{\text{max}}+I_{\text{min}}}$$

where *I_max_, I_min_* represents the largest intensity and minimum intensity of the light respectively.

*γ* = 1 expresses the most ideal condition, which is the biggest contrast.

*γ* = 0 expresses the worst condition, which means no luminance difference between two adjoining points.

**Figure 3.**
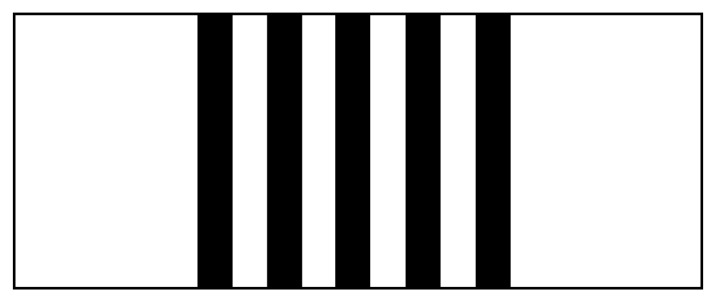
Spatial frequency of a reflecting type Cassegrain telescope.

The spatial frequency, cross axle, means number of the cycles in each millimeter (mm), where each black-and-white pair is called a cycle in Figure 3. A line on the top of this graph, which is a black line with small circles through it, represents the ideal curve.

For a good telescope design the MTF curve comes close to this line. The purple line with the crosses through it shows the on-axis values. The goal is to get this line as high and close to the ideal curve as possible. The violet and the green lines with the triangles and squares through them show the off-axis values. Ideally, these lines should be as good as the on-axis line, and they should be balanced with each other so they are close together [20].

The modulation value which over 0.8 respects to 20 cycles/mm means discrimination or contrast of this design is high and excellent [21]. Hence, we often use MTF to analyze the ability as the resolving power or the sharpness of the telescope. This also makes sure that our designed telescope can obtain clear Sun image outlines.

During design process, we tried to use a plane mirror to replace the right prism and also relocated the right angle prism to a location between the convex mirror and the concave one. For the first case, all the lines separate and are far below the ideal curve, and the spatial frequency only equals 2 when the modulation value equals 0.8. For the other case, only the on-axis curve is close to the ideal curve, and other lines are far away.

## 3. Control Strategies of Sun-Tracking by Image Processing

**Figure 4.**
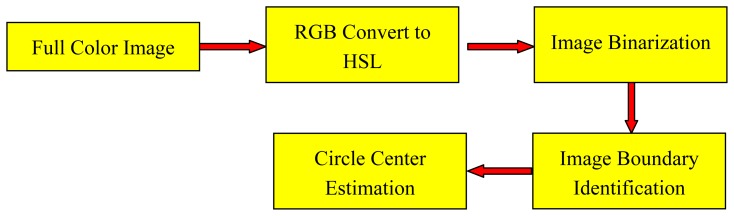
Procedure of estimating the Sun image center with image processing.

An image-based Sun position sensor provides a picture including a Sun image, other objects and inevitable noise. To get a clear and pure Sun image is almost impossible. In order to make a high accuracy Sun tracking system any non-Sun images and noisy points should be distinguished and removed, and therefore a clear Sun image would display. Image processing is usually used to identify the Sun image in the picture. An image processor was proposed in the research that can sufficiently detect Sun images and eliminate any possible noise on the acquired digital picture, as well as accurately find the center of the Sun. The procedures of the proposed image processor are shown in Figure 4.

**Figure 5.**
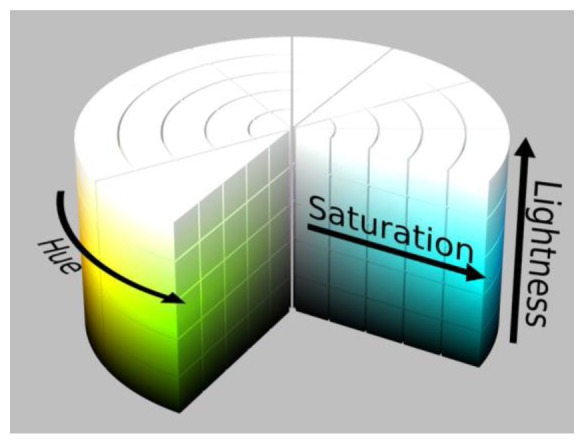
HSL color model.

The color of an object in a digital photo is the combination of Red-Green-Blue (RGB) color elements. RGB is used in many cases but sometimes it's a little difficult to know what color you are describing. HSL is another way to describe a color consisting of hue (the angle on the color wheel), saturation (the amount of chroma) and lightness (how bright the color is), as shown in Figure 5 [22]. Brightness in HSL can be separated to make the colors less vulnerable to the impact of the light intensity, thereby affecting the discrimination of the object.

The formulas for calculating L value is as follows:

(2) $$\text{L}=\frac{1}{2}(\text{max}+\text{min})$$

where r, g and b are normalized to the range of 0∼1; max is the maximum among r, g and b; min is the minimum among r, g and b.

**Figure 6.**
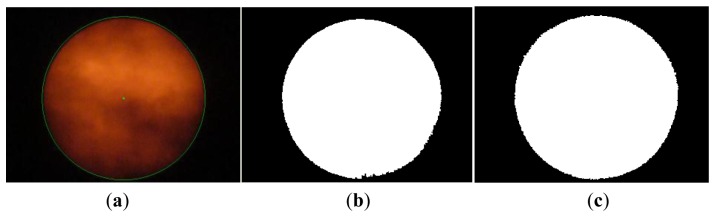
(a) Sun picture of sky with some clouds; (b) Binarization with L: 0.078∼1.0; (c) Binarization with L: 0.039∼1.0.

**Figure 7.**
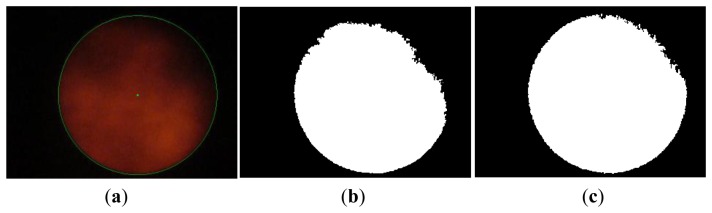
(a) Sun picture of sky with many clouds; (b) Binarization with L: 0.078∼1.0; (c) Binarization with L: 0.039∼1.0.

So as to distinguish Sun images from non-Sun images, image binarization is applied to convert a HSL color photo into black and white by way of setting adequate threshold of lightness. Figures 6(a) and 7(a) show a picture of the sky with some clouds and many clouds, respectively. The binarization of the Sun picture from Figure 6(a) with different lightness thresholds is shown in Figure 6(b and c). The same binarization method was applied to the picture in Figure 7(a) and the results are shown in Figure 7(b and c). Apparently the binary image is more complete using a wider range of thresholds and thus the Sun image is more easily identified.

**Figure 8.**
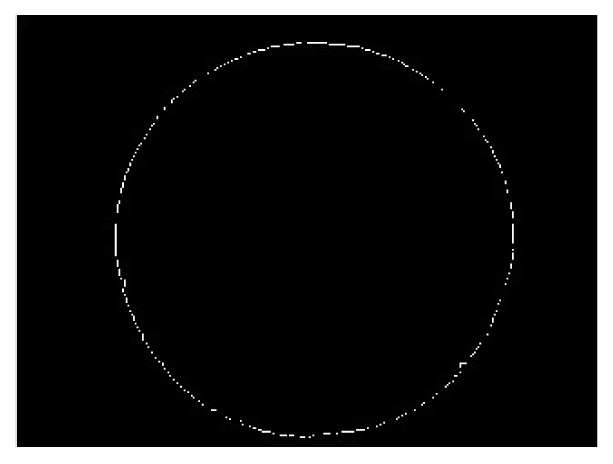
The boundary of the binary image.

After getting a binary Sun image, it is necessary to recognize boundary of the Sun image for finding the center of the Sun. The Sobel method [23] is applied to detect the edge of the Sun image. The edge of the binary image in Figure 6(c) is found by the method and is shown in Figure 8.

The center of the photo frame in the webcam is regarded as the center of the Sun image when the solar tracker completely points to the Sun. Contrarily, the central coordinates of the Sun image are not equal to the oned of the photo frame if the solar tracker deviates from the Sun. Therefore the central coordinates of the Sun image have to be estimated to judge whether the solar tracker is precisely aimed at the Sun. The three-point circle method is used to evaluate the central coordinates (x, y) of the Sun image. Three line segments are determined by choosing three non-collinear arbitrary points on the evaluated circle, and then the intersection of the perpendicular bisectors of the three line segments shall be the center of the circle. It is assumed that the coordinates of three points are (x_1_, y_1_), (x_2_, y_2_) and (x_3_, y_3_). The central coordinates (x, y) is as follows:

(3) $$\begin{array}{cc} x=\frac{b_{2}c_{1}-b_{1}c_{2}}{a_{2}b_{1}-a_{1}b_{2}} & y=\frac{a_{2}c_{1}-a_{1}c_{2}}{a_{1}b_{2}-a_{2}b_{1}} \end{array}$$

where:

$$\begin{array}{ll} a_{1}=x_{1}-x_{2} & a_{2}=x_{1}-x_{3}\\ b_{1}=y_{1}-y_{2} & b_{2}=y_{1}-y_{3}\\ c_{1}=\frac{x_{2}^{2}-x_{1}^{2}+y_{2}^{2}-y_{1}^{2}}{2} & c_{2}=\frac{x_{3}^{2}-x_{1}^{2}+y_{3}^{2}-y_{1}^{2}}{2} \end{array}$$

The algorithm of how to select three points from the edge of Sun image is that the distance between the first point and middle point is same as between the third point and middle point. All points but collinear points on the edge of the Sun image are used to calculate the central coordinates of the Sun image. The central coordinate is determined by the maximum numbers of the same calculated central coordinate in all.

## 4. Experiment Results

**Figure 9.**
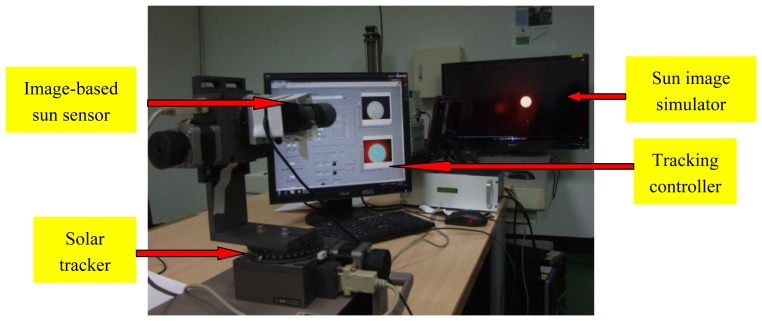
Sun image tracking platform.

In order to test performance of the image-based Sun position sensor and tracking algorithm we established in the laboratory a Sun image tracking platform, shown in Figure 9, which comprises a Sun image simulator and a solar tracking system with our image-based Sun position sensor and tracking controller.

The function of the sun image simulator is to generate simulated solar images. The solar images are taken and recorded by a high resolution camera, with a neutral filter lens (ND400) and infrared filter lens (760 nm) which can get cleaner Sun images, increase the contrast of Sun images, and avoid damage of the camera optical components due to the glare of the Sun. The simulator can represent a true Sun trajectory in the sky.

The image-based Sun position sensor includes an adjustable enlargement telescope (5–15× magnification) and a low resolution (640 × 480, WEICHU WA-306)/high resolution (2,304 × 1,536, Logitech c920) webcam. There are seven experimental cases, *i.e.*, the combination of 6×/9×/12×/15× magnification telescope and high resolution webcam, and 10×/12×/15× magnification telescope and low resolution webcam, for comparison between the various combinations to investigate which combination is better. The resolution of sun position sensor in each combination is shown in Table 1. The combination of high resolution webcam with 15× telescope is better than all others. Its resolution is 0.0017°/pixel. Basically, the higher the resolution is, the better the tracking accuracy is.

**Figure 10.**
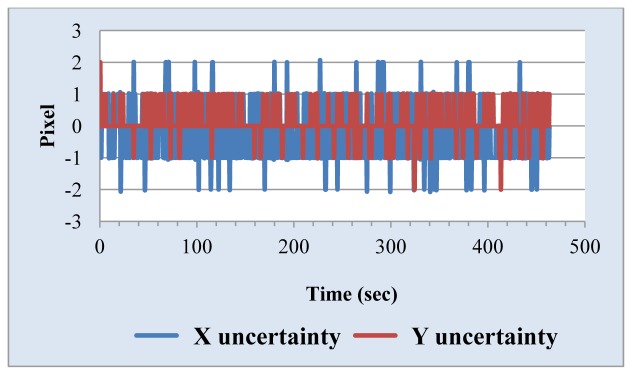
Sensing uncertainty of the Sun position sensor.

**Figure 11.**
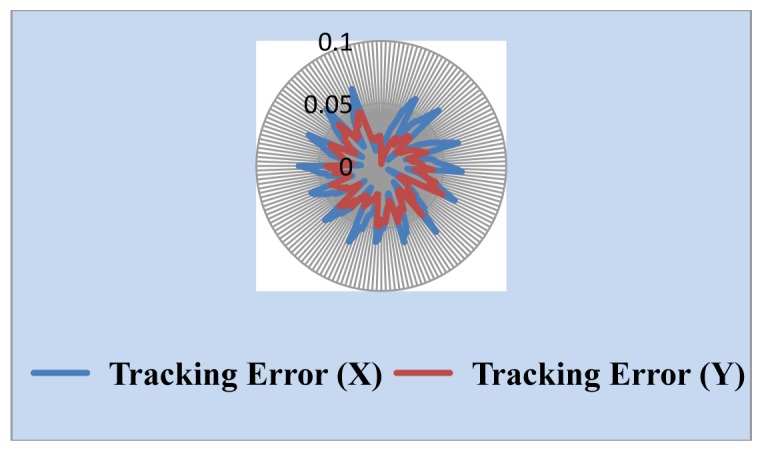
Tracking errors at 10 pixels threshold.

**Figure 12.**
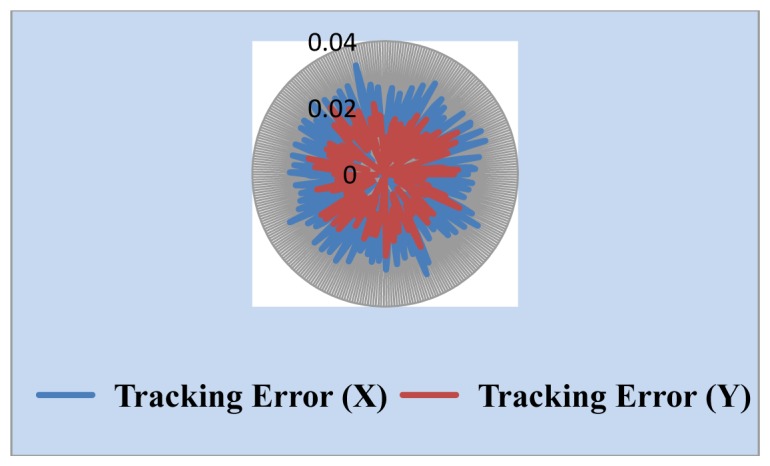
Tracking errors at 5 pixels threshold.

We also measured the uncertainty of the Sun position sensor. The simulated Sun image stopped somewhere and the central coordinates of the Sun image through the solar tracking system was found and is shown in Figure 10. The change of the central coordinates is less than ±2 pixels. One important parameter in the tracking algorithm is the threshold value used for determining whether the solar tracker moves. Generally, the lower the value of the threshold value is, the higher the tracking accuracy is, but it is necessary to think about noise and system uncertainty. The threshold value should not be set too low, or even at zero, because this will make tracking system unstable. We selected the combination of high resolution webcam with a 15× telescope as a Sun position sensor. Threshold value is set to 5 and 10 pixels, respectively. The experimental results, shown in Figures 11 and 12, point out that the tracking error is less than 0.04° in the X coordinate and 0.03° in the Y coordinate, and 0.07° in the X coordinate and 0.05° in the Y coordinate, respectively.

**Figure 13.**
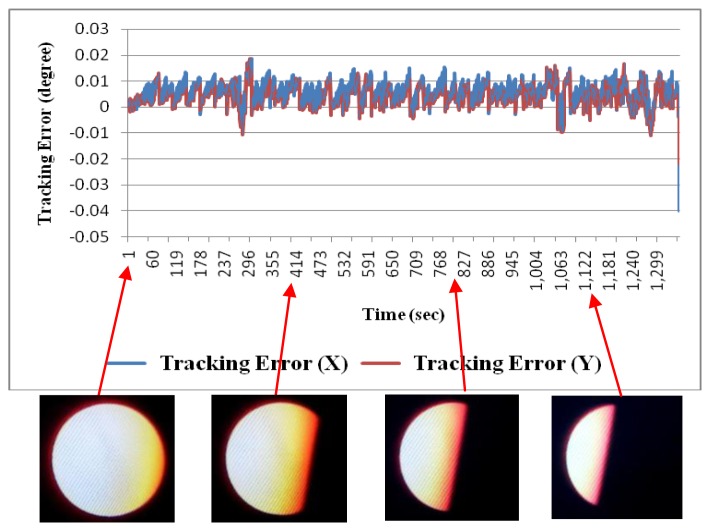
Tracking errors for Sun image shadowing.

In Sun image shadowing experiment the area of the Sun image was shadowed from a little to more than half the Sun. The tracking accuracy was affected and stayed within 0.04°, as shown in Figure 13. This proved that the image-based Sun tracking system has high immunity to different Sun images.

## 5. Conclusions

A scheme and development of a Sun tracking system are introduced in this paper. The tracking system includes a self-designed reflecting type Cassegrain telescope with adjustable magnification, a high resolution webcam and a tracking controller with an embedded image processing algorithm.

By designing a Cassegrain telescope to obtain clear Sun image outlines, the system can offer precise data for the tracking controller to calculate the central coordinates of the Sun images, and then, the tracking controller will send commands to the solar tracker to follow the Sun. Besides, the total length of this design can be held at 10 cm for connection to a webcam, and it also mounts easily on the Sun tracking system due to its light weight.

A Sun image simulator can provide a true Sun trajectory and help test the performance of the image-based Sun position sensor. This article presents a lot of experimental cases and points out the best combination of telescope and webcam for best tracking accuracy. A Sun position sensor composed of a telescope with 15× magnification and high resolution webcam offers the best tracking accuracy and the highest resolution. The tracking accuracy of the solar tracking system is within 0.04° with a window setting of 5 pixels. Undoubtedly, the solar tracking system has good performance on sunny days. Even on cloudy days, it still maintained high tracking accuracy. The Sun image shadowing experiment confirmed this point. Using the image-based Sun position sensor with image processing improves precisely the disadvantages of four-quadrant light sensors and bar-shadow photosensors under low irradiation conditions.

The proposed tracking system scheme is validated on a hardware prototype experimental setup and in an experimental environment. For future work, we will do performance testing in the field and iteratively adjust all parameters of the Sun tracking algorithms to develop an optimal tracking system.

## Figures and Tables

**Table 1.** Sun position sensor resolution in each combination.

	**Telescope Magnification**	**Sun Position Sensor Resolution**
	6×	0.005°/pixel
**Logitech c920**	9×	0.003°/pixel
	12×	0.0025°/pixel
	15×	0.0017°/pixel
	10×	0.006°/pixel
**WEICHU WA-306**	12×	0.005°/pixel
	15×	0.004°/pixel
